# Diagnostic value of non-invasive indices for assessing liver fibrosis in Chinese children with metabolic dysfunction-associated steatotic liver disease

**DOI:** 10.3389/fped.2025.1694863

**Published:** 2026-01-06

**Authors:** Juan Zhou, Min Liu, Jun Qiu, Wenxian Ouyang, Guanghui Zhu, Weijian Chen, Shuangjie Li, Hongmei Zhao

**Affiliations:** 1 Department of Gastroenterology and Nutrition, The Affiliated Children’s Hospital of Xiangya School of Medicine, Central South University (Hunan Children’s Hospital), Changsha, China; 2 Department of Pathology, The Affiliated Children’s Hospital of Xiangya School of Medicine, Central South University (Hunan Children’s Hospital), Changsha, China; 3 Pediatrics Research Institute of Hunan Province, The Affiliated Children’s Hospital of Xiangya School of Medicine, Central South University (Hunan Children’s Hospital), Changsha, China; 4 Department of Hepatology, The Affiliated Children’s Hospital of Xiangya School of Medicine, Central South University (Hunan Children’s Hospital), Changsha, China; 5 Hunan Provincial Key Laboratory of Pediatric Orthopedics, The Affiliated Children’s Hospital of Xiangya School of Medicine, Central South University (Hunan Children’s Hospital), Changsha, Hunan, China

**Keywords:** children, Chinese, liver fibrosis, metabolic dysfunction-associated steatotic liver disease, non-invasive indicators

## Abstract

**Introduction and objectives:**

The rising prevalence of metabolic dysfunction-associated steatotic liver disease (MASLD) in Chinese children necessitates non-invasive fibrosis assessment. However, there is a paucity of pediatric-specific diagnostic tools. The objective of this study was to evaluate the performance of seven established non-invasive indices for staging liver fibrosis in Chinese children with biopsy-proven MASLD.

**Materials and methods:**

A cross-sectional analysis was conducted in children with MASLD. Liver fibrosis staging (F0-F4) was determined via histological assessment. The diagnostic performance of seven non-invasive indices (APRI, FIB-4, PNFI, TyG, AAR, API, NFS) was evaluated using Spearman correlation analysis and area under the receiver operating characteristic curve (AUROC) analysis.

**Results:**

A total of 110 children were included in the study, among whom significant fibrosis (F ≥ 2) was present in 36.3% (40/110), including 12.7% with F3 and 2.7% with cirrhosis (F4). When analyzing the correlations between indices and fibrosis stages, only modest positive correlations were observed for APRI (r = 0.32), FIB-4 (r = 0.23), PNFI (r = 0.24) and TyG (r = 0.24) (all *P* < 0.05), while AAR, API and NFS had no significant correlations (all *P* > 0.05). All indices exhibited poor diagnostic accuracy, with AUROCs ranging from 0.49 to 0.69 across all fibrosis stages. Specifically, APRI, FIB-4 and TyG showed analogous AUROCs (0.60–0.69) for diagnosing any (F ≥ 1), significant (F ≥ 2) and severe (F ≥ 3) fibrosis. Conversely, AAR, API and NFS demonstrated even poorer performance, with AUROCs ranging from 0.49 to 0.60.

**Conclusion:**

The non-invasive fibrosis indices that have been validated demonstrated poor diagnostic accuracy and unreliable performance in Chinese children with MASLD. This diagnostic gap emphasizes the urgent need for the development of pediatric-specific diagnostic algorithms tailored for MASLD-related liver fibrosis.

## 1 Introduction

Metabolic dysfunction-associated fatty liver disease (MASLD), previously termed non-alcoholic fatty liver disease (NAFLD) (1), has emerged as a significant global pediatric health concern with progressively rising prevalence (2). Data reveal notable regional disparities in MASLD prevalence. The obese children in China had a significantly higher prevalence of MASLD than those in Europe (53.4% vs. 38.27%) (3). Liver fibrosis is the most robust prognostic marker for long-term clinical outcomes in MASLD (4, 5) and may progress more rapidly in pediatric patients (6). Therefore, early and precise assessment of liver fibrosis staging in children with MASLD can help improve the clinical outcomes in Chinese children.

Significant diagnostic challenges persist in current pediatric assessment protocols. While liver biopsy remains the gold standard for diagnosing hepatic fibrosis in children, its invasive nature poses potential dangers such as bleeding and infection, which are particularly significant in pediatric populations (7, 8). Crucially, pediatric MASLD exhibits distinct histopathology (e.g., dominant portal inflammation) (9), necessitating age-specific tools. This is partially evidenced by the poor accuracy of adult-oriented models in children, such as Fibrosis 4 score (FIB-4) and aspartate aminotransferase (AST)-to-platelet ratio index (APRI) (10). In light of this, child-specific models have been proposed, such as the pediatric NAFLD fibrosis index (PNFI). However, existing studies on PNFI and other emerging pediatric models remain limited and show controversial performance, underscoring the need for further validation in diverse populations (11). Although international guidelines recommend the use of elastography, factors such as high equipment requirements and lack of standardized operational procedures limit its widespread use in routine clinical practice (12). Therefore, we evaluated seven indices representing diverse origins, including the widely adopted adult scores (APRI, FIB-4, NAFLD fibrosis score (NFS), AST/alanine aminotransferase (ALT) ratio (AAR), albumin platelet index (API)), the metabolism-oriented triglyceride-glucose index (TyG) and the pediatric-specific PNFI, to comprehensively evaluate and compare their diagnostic performance in Chinese children with MASLD.

This study aimed to apply these seven published noninvasive fibrosis scores to Chinese children with biopsy-proven MASLD to evaluate their diagnostic efficacy in fibrosis staging and to explore an optimal noninvasive diagnostic strategy for this population.

## 2 Patients and methods

### 2.1 Study population

This retrospective cross-sectional study evaluated pediatric patients diagnosed with MASLD at Hunan Children's Hospital between January 2012 and December 2023. The diagnosis of MASLD was based on the diagnosis of hepatic steatosis, confirmed by liver biopsy, and the presence of at least one of the following cardiometabolic risk criteria (1): (1) body mass index (BMI) z-scores ≥ 1 standard deviation (SD); (2) fasting blood glucose (FBG) ≥ 5.6 mmol/L; (3) blood pressure ≥ 130/80 mmHg for children aged < 13 years and≥130/85 mmHg for children aged ≥ 13 years; (4) serum triglyceride (TG) concentration ≥ 1.15 mmol/L for children < 10 years and ≥1.70 mmol/L for children ≥ 10 years; (5) high-density lipoprotein cholesterol (HDL-C) concentration ≤ 1.0 mmol/L.

### 2.2 Non-invasive biomarker and evaluation

All participants underwent comprehensive physical examinations and laboratory evaluations. The BMI z-scores were computed by using the World Health Organization Anthro Survey Analyzer software. Liver function parameters, including AST, ALT, gamma-glutamyl transferase (GGT) and serum albumin, were quantified to assess hepatic health. Lipid metabolism was evaluated through measurement of total cholesterol (TC), low-density lipoprotein cholesterol (LDL-C), HDL-C, and TG. Additional hematological and metabolic assessments included a complete blood count with emphasis on platelet count and FBG. All biochemical markers were quantified using commercially available assay kits.

The noninvasive serum biomarker analysis included the AAR, API, APRI, FIB-4, NFS, PNFI and TyG, which were calculated in accordance with published analytical guidelines, as listed in Supplementary Table S1.

### 2.3 Liver histology

Under conscious sedation, an ultrasound-guided percutaneous liver biopsy was performed by the Bard's needle stab method to obtain liver tissue. All biopsy samples were embedded in paraffin blocks after being fixed in 4% neutral formaldehyde. Hematoxylin-eosin and Masson's trichrome were used to stain serial slices (sections were made at intervals of 4 mm). Each liver biopsy sample measured at least 20 mm in length and/or had at least 11 whole portal tracts. Liver biopsies were scored by experienced pathologists according to the NIDDK NASH Clinical Research Network Scoring System.

### 2.4 Portal inflammation and hepatic fibrosis classification

Fibrosis was evaluated using the Scheuer Scoring System, which classifies the extent of fibrotic changes as follows: F0 indicates no fibrosis; F1 denotes fibrous expansion of portal areas; F2 is characterized by periportal fibrosis or portal-portal fibrous septa while maintaining intact liver architecture; F3 represents fibrosis with architectural distortion but without overt cirrhosis; and F4 signifies probable or definite cirrhosis. Any fibrosis was classified as hepatic fibrosis stage ≥ F1; significant fibrosis as hepatic fibrosis stage ≥ F2; severe fibrosis as hepatic fibrosis stage ≥ F3; cirrhosis as hepatic fibrosis stage = F4. Portal inflammation was classified as 0 (none), 1 (mild, sprinkling of lymphocytes in some or all portal tracts), 2 (mild to moderate, denser lymphocytic infiltrate in some or most portal tracts), 3 (moderate to severe, dense lymphocytic infiltrate in most or all portal tracts), or 4 (severe, dense lymphocytic infiltrate in almost all or all portal tracts).

### 2.5 Statistical analysis

We assumed a 20% prevalence of significant fibrosis (≥F2) among patients with MASLD and targeted an AUROC of 0.70 vs. the null AUROC of 0.50, with two-sided *α* = 0.05 and powe*r* = 0.80 (*β* = 0.20). The required total sample size under these assumptions was 105. Our final cohort included 110 biopsy-confirmed patients with MASLD, thus satisfying this requirement. The Kolmogorov–Smirnov test was used to assess the normality of continuous data. The data were expressed as the mean ± standard deviation (SD), or in the range of the median (quartile), as appropriate. Spearman's analysis was used to ascertain the correlation between non-invasive indicators and fibrosis stages.

The diagnostic performance of the non-invasive indicators in identifying the stage of liver fibrosis was evaluated by receiver operating characteristic curve (ROC) and AUROC calculation using liver biopsy as the reference standard. The diagnostic test was deemed to be of excellent quality when the AUROC exceeded 0.80, and of clinical utility when the AUROC ranged between 0.70 and 0.80. All tests were two-tailed, and *P* < 0.05 was considered to be statistically significant.

## 3 Results

### 3.1 Characteristics of children with MASLD

A total of 121 individuals were included. There are 2 cases of chronic liver disease due to other etiologies (viral, autoimmune, genetic), 3 cases of liver injury due to alcohol or drugs, and 6 cases with incomplete clinical data were excluded. The final analysis included 110 patients with biopsy-confirmed MASLD (Supplementary Figure S1). Table 1 provides a synopsis of anthropometric, clinical, laboratory, and histological characteristics of children with MASLD. Notably, a marked male predominance was observed (97.3%, *n* = 107/110), which may limit generalizability to females. The average age of the children was 11.10 ± 1.76 years. Portal inflammation (score ≥1) was observed in 109 (99.1%) of the children. The stage of fibrosis was as follows: 18 (16.4%) in F0, 52 (47.3%) in F1, 23 (20.9%) in F2, 14 (12.7%) in F3, and 3 (2.7%) in F4.

**Table 1:** Characteristics of the participants.

Characteristic	Overall (*n* = 110)
Age (year)	11.10 ± 1.76
Male (*n*, %)	107 (97.3)
BMI z-scores	2.60 (2.06, 2.93)
WHtR	0.57 ± 0.05
SBP (mmHg)	112.38 ± 12.74
DBP (mmHg)	69.29 ± 10.24
ALT (U/L)	133.40 (82.60, 217.70)
AST (U/L)	67.60 (44.40, 94.92)
GGT (U/L)	47.50 (30.08, 72.22)
Albumin (g/L)	44.77 ± 5.16
PLT(10^9^/L)	314.41 ± 80.05
FBG (mmol/L)	5.05 ± 1.42
TC (mmol/L)	4.32 ± 0.89
TG (mmol/L)	1.60 (1.19, 2.30)
HDL-C (mmol/L)	1.09 ± 0.28
LDL-C (mmol/L)	2.62 ± 0.72
Portal inflammation (*n*, %)	
Score 0	1 (0.9)
Score 1	86 (78.2)
Score 2	21 (19.1)
Score 3	2 (1.8)
Score 4	0 (0)
Fibrosis stage (*n*, %)	
F0	18 (16.4)
F1	52 (47.3)
F2	23 (20.9)
F3	14 (12.7)
F4	3 (2.7)

Data are reported as mean ± standard deviation, median and interquartile ranges. ALT, alanine aminotransferase; AST, aspartate aminotransferase; BMI, body mass index; DBP, diastolic blood pressure; FBG, fasting blood glucose; GGT, gamma-glutamyl transferase; HDL-C, high-density lipoproteins; LDL-C, low-density lipoproteins cholesterol; PLT, platelet count; SBP, systolic blood pressure; TC, total cholesterol; TG, triglycerides; WHtR, waist-to-hip ratio.

### 3.2 Association between non-invasive indices and liver fibrosis stages

**Figure 1:**
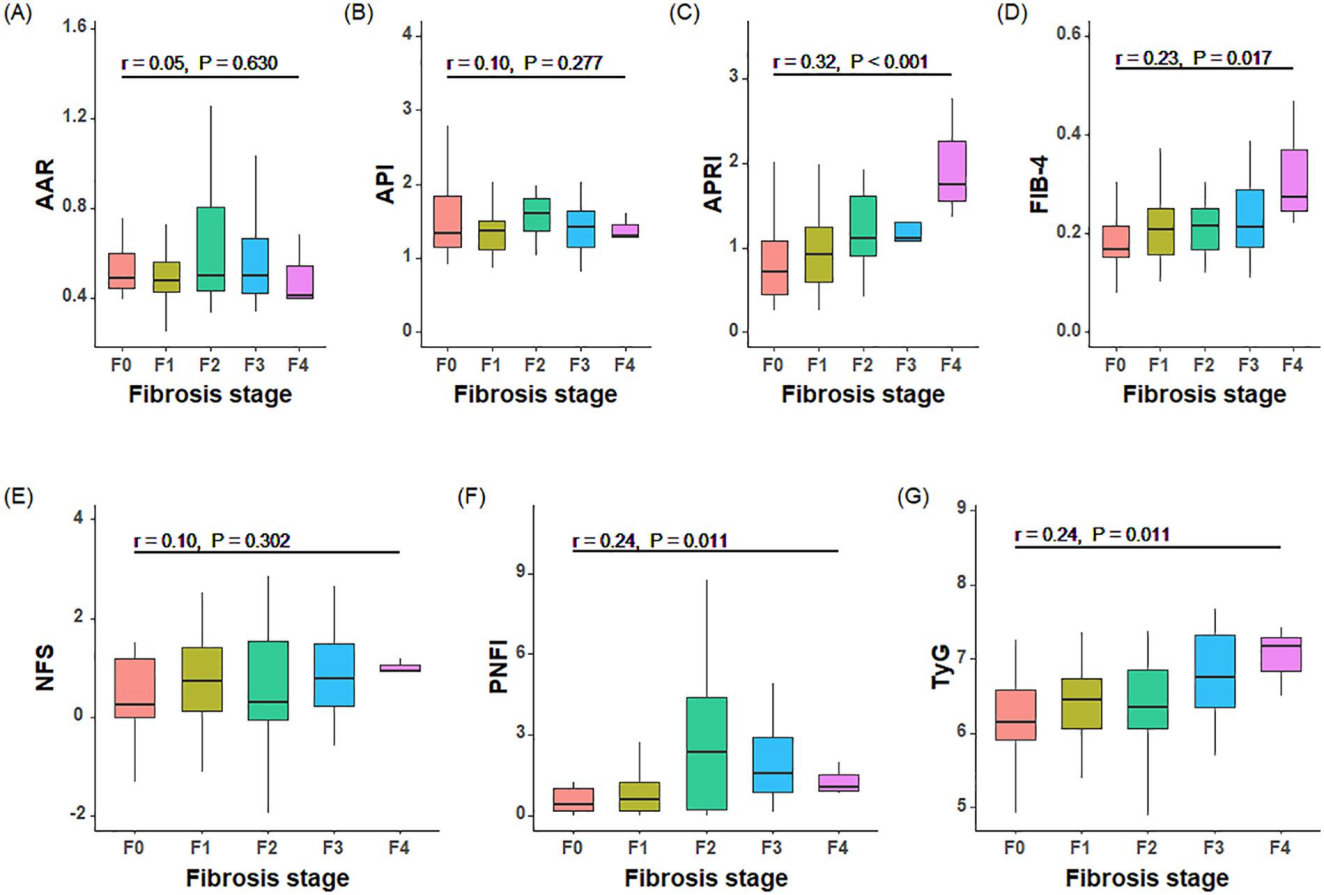
Correlation between non-invasive indicators and liver fibrosis stages. **(A)** AAR, AST to ALT ratio; **(B)** API, albumin platelet index; **(C)** APRI, aspartate aminotransferase to platelet ratio index; **(D)** FIB-4, Fibrosis-4 score; **(E)** NFS, NAFLD fibrosis score; **(F)** PNFI, paediatric NAFLD fibrosis index; **(G)** TyG, triglyceride-glucose. Spearman correlation coefficients (r) and P values are shown in each panel.

Figure 1 shows the correlation of non-invasive indicators and liver fibrosis stages. In children diagnosed with MASLD, the APRI, FIB-4, PNFI and TyG levels were found to be positively correlated with the hepatic fibrosis stage. The Spearman correlation coefficients for APRI, FIB-4, PNFI and TyG were 0.32, 0.23, 0.24 and 0.24, respectively (all *P* values < 0.05). The present study found no evidence to support the hypothesis that there is a link between liver fibrosis stages and AAR, API, as well as NFS (all *P* values > 0.05).

### 3.3 Diagnostic performance of non-invasive indices for fibrosis staging

**Figure 2:**
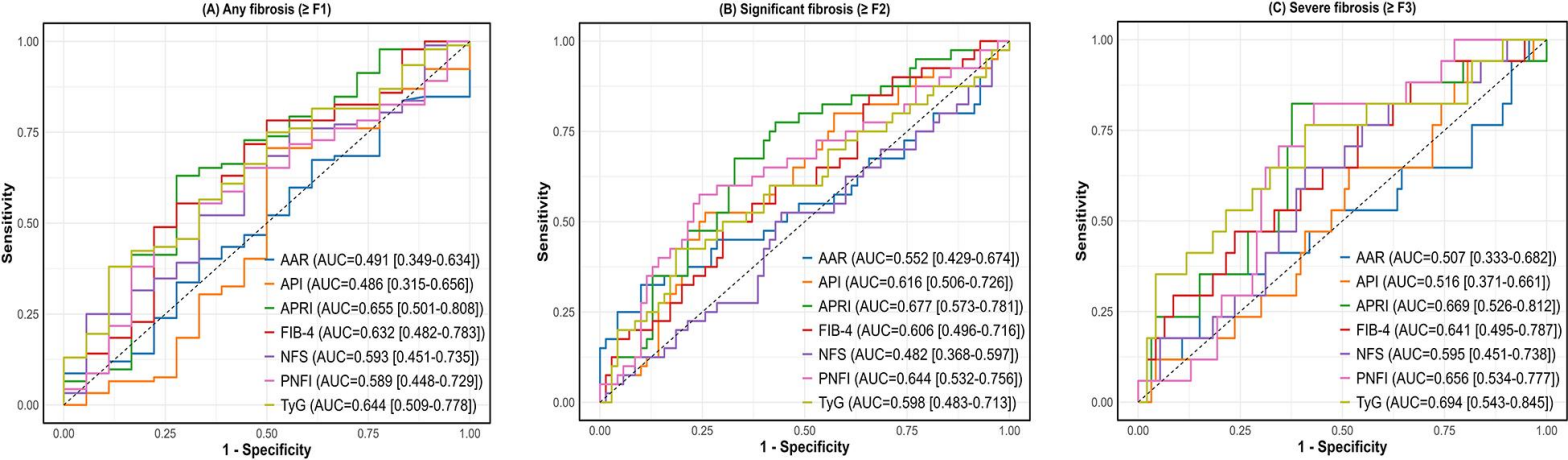
ROC curves of different stages of liver fibrosis in children with MASLD. **(A)** ROC curve of any fibrosis (≥F1) in children with MASLD; **(B)** ROC curve of significant fibrosis (≥F2) in children with MASLD; **(C)** ROC curve of severe fibrosis (≥F3) in children with MASLD; MASLD, Metabolic dysfunction-associated fatty liver disease; ROC, Receiver operating characteristic curve.

The ROC curves and AUROC of non-invasive indicators of different stages of liver fibrosis are shown in Figure 2. Existing non-invasive indicators performed less well than ideal for the classification of fibrosis in children, with an AUROC under 0.70. The ROC curve of the invasive indicators, including APRI, FIB-4 and TyG had similar AUROC in the diagnosis of any fibrosis (F ≥ 1), significant fibrosis (F ≥ 2), and severe fibrosis (F ≥ 3), ranging from 0.60 to 0.69. Furthermore, the predictive value of AAR, API and NFS for hepatic fibrosis was found to be weak, with AUROC values ranging from 0.49 to 0.60.

## 4 Discussion

In this study conducted on the pediatric population, we found that existing non-invasive markers exhibit limited diagnostic efficacy for liver fibrosis in children with MASLD. Although a significant positive correlation was observed between liver fibrosis staging and the APRI, FIB-4, PNFI, and TyG indices, the strength of these correlations remained weak. The AUROC values for all indices were below 0.70, indicating insufficient ability to differentiate between varying stages of liver fibrosis in children.

In studies of adult MASLD populations, the FIB-4 and NFS scores are commonly employed as initial screening tools for assessing liver fibrosis (13). A meta-analysis of 64 studies involving 13,046 patients demonstrated high diagnostic accuracy of FIB-4 and NFS for advanced fibrosis, with an AUROC of 0.84 in adults (14). These indices stratify patients into low-, intermediate-, and high-risk groups for advanced fibrosis using validated cut-offs (FIB-4: <1.30 and >2.67; NFS:<-1.455 and >0.676), though accuracy varies with age and transaminase levels (15). Critically, this adult-based framework does not translate effectively to pediatric cohorts. Alkhouri et al. (16) reported that both FIB-4 and NFS produced AUROCs <0.70 for predicting advanced fibrosis in a biopsy-proven sample of 242 children with MASLD, which is consistent with our results. Similarly, it was shown that AAR, API, and APRI could not detect any, significant or advanced fibrosis in children with MASLD. The potential for these discrepancies to be attributable to the study population. First, it should be noted that the laboratory indices involved in these non-invasive indicators (e.g., platelet and albumin levels) follow age-specific developmental trajectories, which may reduce the applicability of adult-derived formulas to pediatric populations. Second, histological features should be carefully considered. Pediatric MASLD predominantly exhibits zone-1 (periportal) inflammation, observed in 99% of cases in our study, often accompanied by periportal fibrosis (17). This contrasts with the typical zone 3 (centrilobular or pericentral) steatosis and fibrosis seen in adults, where portal inflammation occurs in only 82% of cases (18). Given these age-related biochemical variations and distinct histopathological patterns, adult-derived non-invasive fibrosis indices may fail to accurately reflect the pathophysiological processes in pediatric MASLD, underscoring the need for pediatric-specific diagnostic algorithms.

In this study, TyG showed only a weak correlation with fibrosis and exhibited suboptimal diagnostic performance. As an indicator of insulin resistance (19), TyG has recently been reported in a cross-sectional study of obese children and found to correlate weakly with MASLD, and the authors concluded its predictive value in pediatric populations remains unestablished (20). Similarly, Arata et al. observed that insulin resistance was associated with hepatic steatosis but not with fibrosis in children (21). A plausible explanation is that, compared with adults, insulin resistance in children may induce hepatocellular injury and inflammation at an earlier disease stage, prior to substantial fibrotic remodeling. Consequently, indices such as TyG, which primarily reflect insulin resistance, may be insufficient to capture these early hepatic alterations. However, it should be noted that PNFI, previously reported by Nobili et al. to hold good predictive value, did not replicate those findings in our study (22). Our results align with those of Draijer LG et al. (23), who conducted the first systematic review on the diagnostic accuracy of fibrosis tests in pediatric MASLD. That analysis included 20 studies totaling 1,787 subjects and assessed multiple markers, including TyG, APRI, FIB-4, API, and PNFI. The authors concluded that, due to inadequate validation, the accuracy and clinical utility of noninvasive fibrosis testing in children with MASLD remain uncertain. Emerging evidence in adults indicates that combining biochemical and imaging-based markers, including the enhanced liver fibrosis score and the FibroScan-AST score, significantly enhances diagnostic accuracy for MASLD (24, 25). Future pediatric studies should evaluate the feasibility and validity of such multi-parameter approaches to improve fibrosis detection and risk stratification in children.

The presence of liver fibrosis represents the most clinically relevant predictor of long-term liver-related events and overall mortality. This association persists even in early fibrosis stages, with adverse events increasing progressively as fibrosis advances (6). In our study, liver fibrosis was predominantly at stage F1 (47.3%), with only 2.7% at stage F4, reflecting the generally mild degree of fibrosis in pediatric MASLD. Under such conditions, the capacity to differentiate between absent and any fibrosis (F ≥ 1) becomes critical for guiding therapeutic decisions and evaluating intervention efficacy. However, the findings of this study indicate that laboratory-based non-invasive indices perform poorly in identifying mild-to-moderate fibrosis, posing a significant challenge to current clinical practice. Clinicians should interpret existing non-invasive markers cautiously when applied to children with MASLD and avoid overreliance on them for fibrosis staging. Additionally, 97.3% of the patients in this study were male, consistent with the epidemiological data showing higher MASLD prevalence in boys. Further investigation is needed to determine whether this marked sex imbalance influences the performance of non-invasive fibrosis indices.

The principal strength of this study is the confirmation of all MASLD cases by liver biopsy. Compared with liver fibrosis assessments using elastography or the NFS, these findings provide stronger evidence for the association between non-invasive indices and liver fibrosis in pediatric patients with MASLD. This study has several limitations. First, although the demographic profile reflects regional referral patterns, generalizability to broader, multiethnic pediatric populations with balanced sex distribution is limited by the single-center design and male predominance. Second, the retrospective cross-sectional design precludes evaluation of long-term predictive value. Third, modest sample sizes restricted the robustness of multivariable and stratified analyses, particularly after adjustment for metabolic covariates. These limitations highlight the need for prospective, multicenter studies with sufficient sample sizes to recalibrate or refine pediatric-specific fibrosis indices and validate their prognostic performance.

The findings of this study indicate that the data presented do not support the accuracy of non-invasive indices, including AAR, API, APRI, FIB-4, NFS, PNFI, and TyG, for the detection of hepatic fibrosis in children with MASLD. Future studies should prioritize the development and validation of pediatric-specific diagnostic algorithms that could incorporate clinical, biochemical, and imaging parameters to enable accurate and non-invasive assessment of liver fibrosis in children.

## Data Availability

The raw data supporting the conclusions of this article will be made available by the authors, without undue reservation.
