# Supplementary material for: Diagnostic value of non-invasive indices for assessing liver fibrosis in Chinese children with metabolic dysfunction-associated steatotic liver disease
Source: Front Pediatr. 2026 Jan 6;13:1694863. doi: 10.3389/fped.2025.1694863 (PMC12816359; doi:10.3389/fped.2025.1694863)
Supplement: Supplementary file 1 [file Supplementaryfile1.docx]

**Supplementary Table S1.** Overview of tested noninvasive fibrosis scores.

| **Score** | **Mathematical Formula** |
| --- | --- |
| AAR | $\frac{\text{AST (IU/L)}}{\text{ALT (IU/L)}}$ |
| API | $\frac{\text{Albumin (g/dL) × PLT (10}^{9}\text{/L)}}{1000}$ |
| APRI | $\frac{\text{AST (IU/L) / upper level of AST (IU/L)}}{\text{PLT (10}^{9}\text{/L)}}\times100$ |
| FIB-4 | $\frac{\text{Age (years) × AST (IU/L)}}{\text{PLT (10}^{9}\text{/L) }\times\text{ }\sqrt{\text{ALT (IU/L)}}}$ |
| NFS | *$\text{p1}=\text{-1.675 + 0.037 }\times\text{ }A\text{ge }(\text{years})\text{+ 0.094 }\times\text{ BMI }(\text{kg/m}^{2})$*  *$\text{p}2=1.13\times\text{Diabetes }(\text{yes}=1\text{，no}=0\text{）}$*  *$\text{p3}=\text{ 0.99 }\times\text{ }\frac{AST(IU/L)}{ALT (IU/L)}\text{ -0.013 }\times\text{ }P\text{LT }(10^{9}\text{/L})\text{ -0.66}\times\text{ }A\text{lbumin}(\text{g/dL})$*  *$\text{NFS}=\text{p1}+\text{p2}+\text{p3}$* |
| PNFI | $\text{p = - 6.539 }\times\text{ ln(Age [years]) + 0.207(WC [cm]) + 1.957 }\times\text{ ln(TG [mg/dL]) - 10.074}$ $\text{PNFI = }\frac{\text{EXP(p)}}{\text{1+EXP(p)}}\text{ }\times10$ |
| TyG | $\text{ln (}\frac{\text{TG [mg/dL] × FBG [mg/dL]}}{2}\text{)}$ |

AAR, AST to ALT ratio; ALT, alanine aminotransferase; API, albumin platelet index；APRI, aspartate aminotransferase to platelet ratio index; AST, aspartate amino transferase; EXP, exponential; FIB-4, Fibrosis-4 score; NFS, NAFLD fibrosis score; PNFI, paediatric NAFLD fibrosis index; TyG, triglyceride-glucose index.


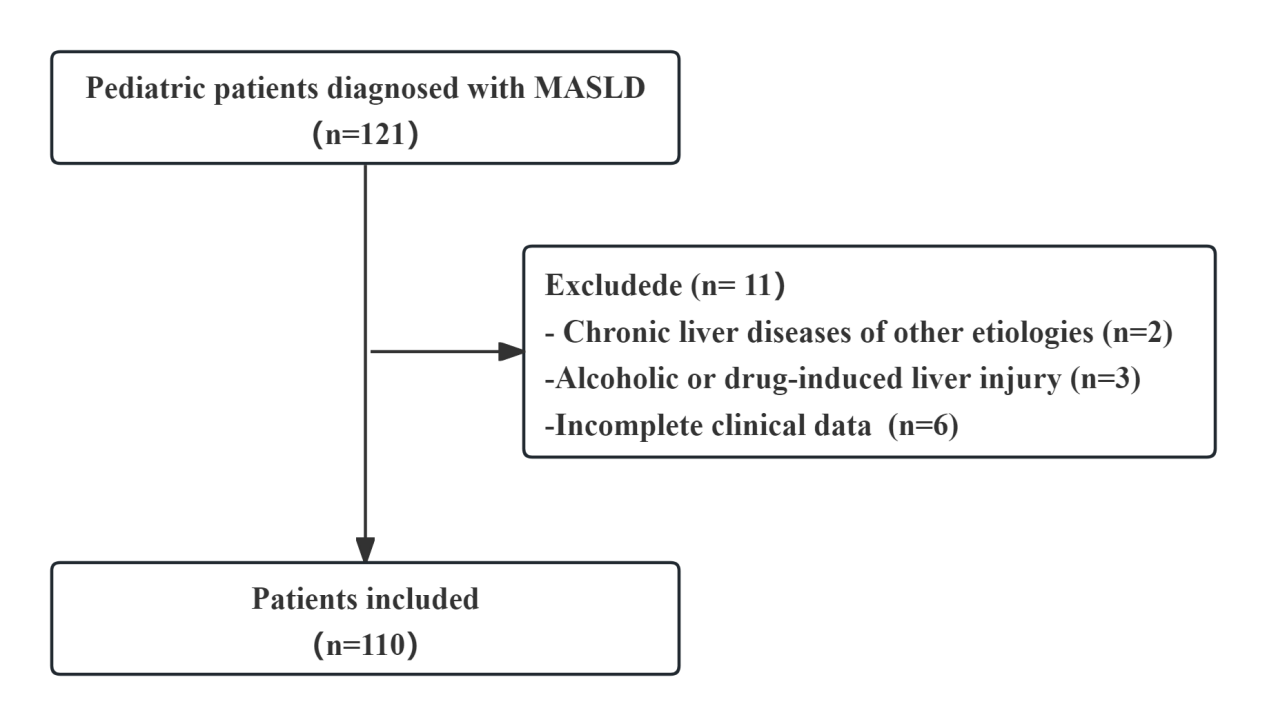


**Supplementary Figure S1.** Flow diagram of study population
